# Cardiometabolism as an Interlocking Puzzle between the Healthy and Diseased Heart: New Frontiers in Therapeutic Applications

**DOI:** 10.3390/jcm10040721

**Published:** 2021-02-12

**Authors:** Teresa Pasqua, Carmine Rocca, Anita Giglio, Tommaso Angelone

**Affiliations:** 1 Department of Health Science, University Magna Graecia of Catanzaro, 88100 Catanzaro, Italy; teresa.pasqua@unicz.it; 2 Laboratory of Cellular and Molecular Cardiovascular Pathophysiology, Department of Biology, E. and E.S. (Di.B.E.S.T.), University of Calabria, 87036 Rende (CS), Italy; 3 Department of Biology, E. and E.S. (Di.B.E.S.T.), University of Calabria, 87036 Rende (CS), Italy; anita.giglio@unical.it; 4 National Institute of Cardiovascular Research (I.N.R.C.), 40126 Bologna, Italy

**Keywords:** cardiometabolism, cardiac physiology, heart failure, ischemia, therapeutic targets

## Abstract

Cardiac metabolism represents a crucial and essential connecting bridge between the healthy and diseased heart. The cardiac muscle, which may be considered an omnivore organ with regard to the energy substrate utilization, under physiological conditions mainly draws energy by fatty acids oxidation. Within cardiomyocytes and their mitochondria, through well-concerted enzymatic reactions, substrates converge on the production of ATP, the basic chemical energy that cardiac muscle converts into mechanical energy, i.e., contraction. When a perturbation of homeostasis occurs, such as an ischemic event, the heart is forced to switch its fatty acid-based metabolism to the carbohydrate utilization as a protective mechanism that allows the maintenance of its key role within the whole organism. Consequently, the flexibility of the cardiac metabolic networks deeply influences the ability of the heart to respond, by adapting to pathophysiological changes. The aim of the present review is to summarize the main metabolic changes detectable in the heart under acute and chronic cardiac pathologies, analyzing possible therapeutic targets to be used. On this basis, cardiometabolism can be described as a crucial mechanism in keeping the physiological structure and function of the heart; furthermore, it can be considered a promising goal for future pharmacological agents able to appropriately modulate the rate-limiting steps of heart metabolic pathways.

## 1. Introduction

**Figure 1:**
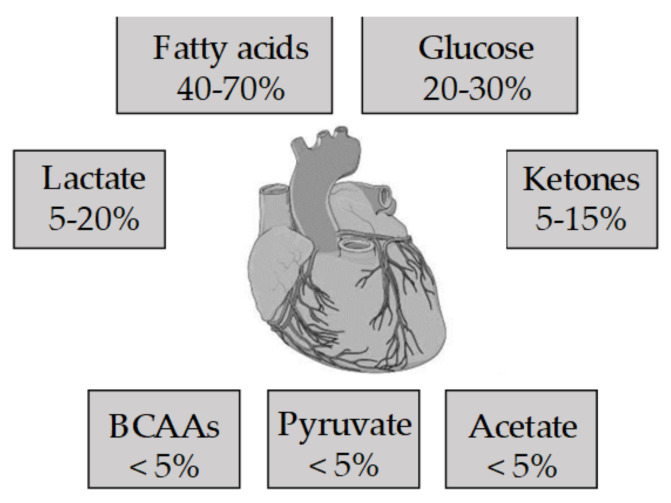
Metabolic substrate used by the healthy heart. BCAAs, branched-chain amino acids.

For the first time in 1878, with his lecture entitled “On the nutrition of the frog heart” [1], the physiologist Hugo Kronecker introduced to the scientific scenario the concept of cardiac metabolism. During the last century, extensive evidence has contributed to define metabolism as an important milestone of cardiac physiology correlating structure to function. Indeed, the heart works as a chemo-mechanical transducer able to use oxygen and metabolic substrates to sustain its own contractile activity. Unlike the anoxia- and hypoxia-tolerant hearts, such as frog and turtle hearts, which have large glycogen stores, many mammalian hearts, including the human heart, can rely only on a limited storage of endogenous content of high-energy phosphate that would be sufficient to support heart function only for a very short time. This highlights the importance of a finely regulated metabolism for the maintenance of cardiac homeostasis [2]. Under physiological conditions, the heart can be considered an omnivore organ due to the wide range of energy-substrates it uses [3] (Figure 1). Generally, the used substrate choice depends on the plasma levels, on the specific transporters localized on the cardiomyocyte membrane, and on the activity of crucial enzymes/modulators that control the energetic pathways [1,4,5]. However, regardless of the initial substrate, each molecule converges to acetyl-coenzyme A and to the generation of NADH and FADH2 (reducing equivalents). This contributes to the electron transport chain, creating the proton gradient necessary for the activity of F0F1-ATP/synthase and for the production of adenosine triphosphate (ATP) [3]. Of note, cardiac metabolism is also importantly regulated by the levels of intermediary metabolites belonging to the pathways [3].

The myocardial metabolic activity takes place within the cardiomyocytes. Here, a very large number of mitochondria carry out the oxidative phosphorylation of adenosine diphosphate (ADP) to approximately generate the 95% of ATP used as cardiac energy source [2]. This source is maximized through highly integrated enzyme pathways [6], which allow a quick and well-organized response regulated at different levels [1]. ATP in the heart can be produced from different energetic substrates, such as carbohydrates, fatty acids, amino acids and ketone bodies (Figure 1) [3]. The choice of the used substrates strictly depends on cardiac work: an acute rise in cardiac workload predominantly induces the mobilization of carbohydrates, while under normal conditions, the heart mainly draws from fats [7]. Moreover, cardiac metabolism also depends on complex regulatory pathways, including allosteric, transcriptional and post-transcriptional modulations [2,8,9,10].

Presently, it is widely recognized that cardiac metabolism deeply influences heart physiopathology, whose balance, under different stimuli, strictly depends on the flexibility of the metabolic networks. Moreover, the cardiac muscle itself is able, particularly in the presence of chronic diseases, to remodel its metabolism in order to preserve its function. In this perspective, the present review aims to provide an overview regarding the metabolic changes detectable in the heart under pathological conditions, focusing on the main observed alterations and at the same time underlining possible therapeutic strategies.

To better deepen the topic, a brief description of the most important cardiac metabolic pathways is mandatory.

## 2. The Metabolism of the Healthy Heart

### 2.1. Cardio Metabolism of Glucose

The first step of carbohydrate metabolism consists of glucose uptake by cardiomyocytes via the action of transporters (Glucose Transporters family, GLUTs). These proteins generally respond to the Michaelis–Menten kinetics [11] and represent a rate limiting step for the substrate utilization in the myocardium [12]. The heart expresses mostly the insulin-independent glucose transporter isoform (GLUT-1), and the insulin-sensitive isoform GLUT-4 [13].

**Figure 2:**
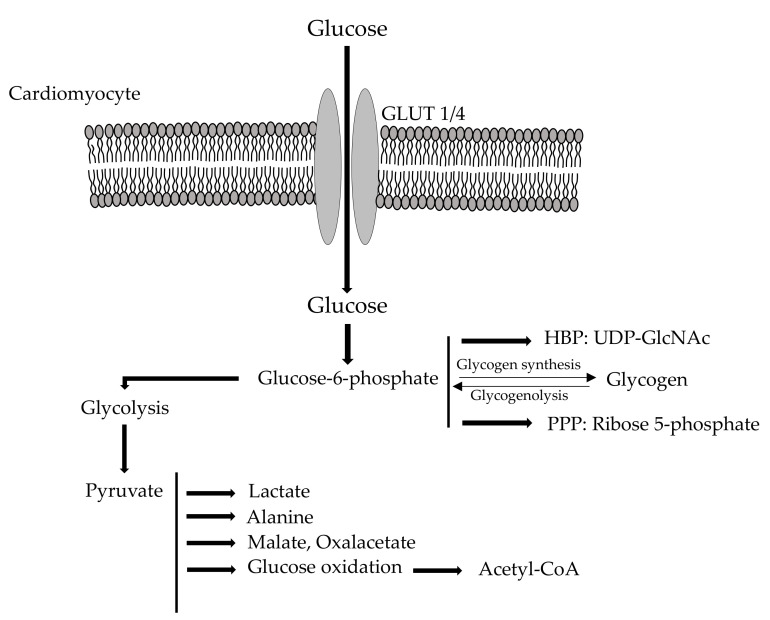
Cardiometabolism of glucose. GLUT 1/4: Glucose Transporter; HBP: Hexosamine biosynthetic pathway; UDP-GlcNAc: uridine diphosphate-N-acetylglucosamine; PPP: Pentose phosphate pathway.

Within the cardiac cells, glucose is then converted by hexokinase, a rate limiting enzyme for glycolysis [14], into glucose-6-phosphate that can undergo one of the following pathways [1] (Figure 2).

**Synthesis of glycogen** represents the main cell deposit form for glucose. The real metabolic role of glycogen within the cardiac muscle is still debated. It has been demonstrated that glycogen, together with glycogen phosphorylase, associates with the sarcoplasmic reticulum [15] and with the contractile function [16]. Of note, the fetal heart is extremely rich in glycogen, presumably to provide energy during the hypoxic phase of birth [17]. Glycogen synthesis is regulated by both covalent modification and allosteric regulators [1].

**Glycolytic pathway** represents the way in which glucose provides a small amount of ATP in the heart. The rate-limiting step of the cycle is the enzyme phospho-fructokinase-1 (PFK-1), characterized by an intricate allosteric regulation [18,19,20]. Fructose 1,6-bisphosphate, adenosine monophosphate (AMP), and fructose 2,6-bisphosphate are positive modulators, while protons, citrate, and ATP are negative allosteric modulators [18,19,21]. If PFK is highly activated, as in the case of increased cardiac work or ischemia [22,23], glycolysis is also regulated at the level of triose-phosphate dehydrogenase [1].

**Pentose phosphate pathway (PPP)**—In this case glucose is conveyed to the synthesis of purine and of reducing equivalents. In particular, glucose 6-phosphate (G6P) can undergo two different pathways, i.e., oxidative and irreversible, or non-oxidative and reversible [24]. The non-oxidative branch plays an important role in the synthesis of sugar phosphates by interconverting glycolytic intermediates though transaldolase [24]. On the other hand, the oxidative mechanism gives rise to the production of ribulose 5-phosphate and NAPDH. The conversion of G6P to 6-phosphogluconolactone by glucose 6-phosphate dehydrogenase (G6PDH) represents the rate-limiting step [1]. G6PDH is inhibited by increased levels of NADPH and de-inhibited by increased levels of NADP+ or oxidized glutathione [25,26].

**Hexosamine biosynthetic pathway (HBP)**—Glucose acts as a precursor for the conversion of fructose 6-phosphate to uridine diphosphate-N-acetylglucosamine (UDP-GlcNAc), which is then used for protein modifications [27]. The HBP rate-limiting step is represented by fructose 6-phosphate amidotransferase (GFAT). GFAT, using the amino group provided by glutamine, produces glucosamine by the transamination of fructose 6-phosphate [27,28]. GFAT regulation may depend on phosphorylation by PKA, or on transcriptional mechanisms [29], or on UDP-GlcNAc negative feedback [27].

At this point, another crucial glycolytic actor is pyruvate, which can be transformed into lactate, alanine, oxaloacetate, malate, or more importantly, acetyl-CoA [1,30,31,32]. Physiologically, pyruvate is transferred by a specific transporter into the mitochondrion, where it is carboxylated to oxaloacetate or decarboxylated to acetyl-CoA [32,33]. The last reaction is strictly regulated by the pyruvate dehydrogenase complex (PDC) [34], which can be, in turn, modulated by phosphorylation (inactivation) and dephosphorylation (activation) [35]. In general, ATP, acetyl-CoA, lactate and NADH are kinase activators, while ADP, CoA SH, pyruvate and NAD+ inhibit these enzymes [1]. An increase in acetyl-CoA is also able to mediate PDC inhibition due to the oxidation of fatty acids or ketone bodies [36]. On the contrary, when cardiac work increases, a reduction of acetyl-CoA, ATP and NADH can be observed, leading to PDC activation [37].

### 2.2. Myocardial Fatty Acid Metabolism

**Figure 3:**
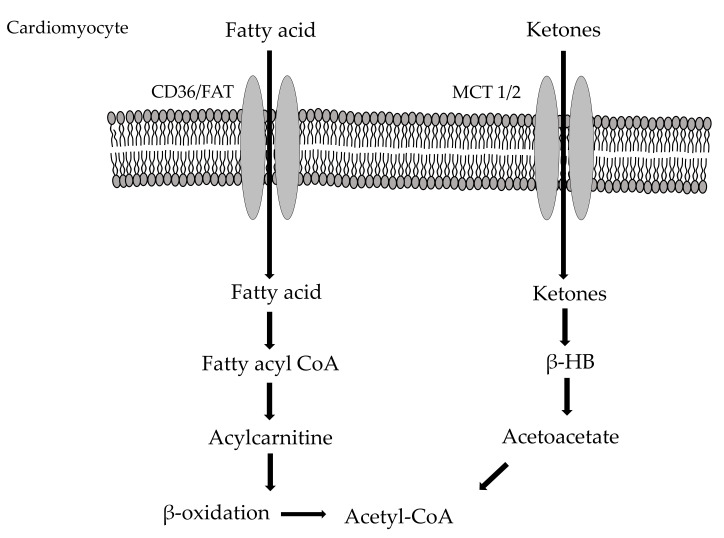
Cardiometabolism of fatty acids and ketones. CD36/FAT: Fatty Acid Transport proteins; MCT 1/2: Monocarboxylate Transporter; β-HB: β-hydroxybutyrate.

Long-chain fatty acids are the main energy source for heart respiration, their β-oxidation being the first cardiometabolic cascade to be identified [38]. This pathway is regulated by changes in acyl-CoA, FAD+ and NAD+ concentrations, reflecting the effective workload or oxygenation of the myocardium [39]. Saturated fatty acid palmitate and monounsaturated long chain fatty acid oleate are the most abundant fatty acids detectable in the human blood stream [1]. In particular, long chain fatty acids derive from triglycerides and are lastly addressed to the Krebs cycle as acetyl-CoA. Fatty acids enter the cardiac cells due to protein carriers known as heart-specific fatty acid-binding protein (h-FABP) [40]. Here they are converted into fatty-acyl coenzyme A and then transported into the mitochondria where β-oxidation takes place [41]. This translocation represents the main rate-limiting step of the entire metabolic pathway, and starts with the shift of an acyl group from acyl CoA to carnitine through carnitine palmitoyl transferase I (CPT-I) [1]. This enzyme is inhibited by a product of acetyl-CoA carboxylation, namely, malonyl-CoA, produced by the action of acetyl-CoA carboxylase (AAC) and decarboxylated by malonyl-CoA decarboxylase (MCD) [42,43,44,45]. In the heart, AMP kinase phosphorylates and inhibits ACC, lowering malonyl-CoA and increasing oxidation of fatty acids [46]; conversely, when fatty acids increase, peroxisome proliferator-activated receptor α (PPARα) induces the expression of MCD [45]. Moreover, several experimental evidences demonstrated that both ACC and MCD play a crucial role in cardiac physiological metabolism [2,44,47]. Once in the mitochondria, carnitine acyl-CoA transferase II (CPT-II) moves the carnitine-acyl unit to CoA SH; then, acyl-CoA is conveyed to β-oxidation (Figure 3).

Cardiac fatty acid utilization is augmented when plasma fatty acid levels increase, causing a general raise in the expression of related proteins, such as UPC3 [48,49,50]. This mechanism is mediated by the nuclear receptor PPARα; one of its most important cofactors is represented by PPAR-γ coactivator 1α (PGC-1α) that, in turn, is importantly involved in the biogenesis of mitochondria [51,52,53].

### 2.3. Cardio Metabolism of Ketone Bodies/Amino Acids

Even if to a lesser extent, amino acids and ketone bodies participate to the energy metabolism of the heart [54]. Amino acids are interested by the action of cardiac transaminases and provide important substrates for the Krebs cycle [55]. In addition, some amino acids also contribute to the mitochondrial electron transport chain, needed for the oxidation of cytosolic NADH [56]. On the other hand, ketone bodies are relevant especially under exercise, fasting, and during heart failure (HF), when their plasma levels increase significantly promoting their entry in the Krebs cycle [1] (Figure 3). Since both the amino acid and ketone body availability is very low under physiological conditions, they are considered to give a small contribution to the total oxidative metabolism of the heart [57].

## 3. Cardiac Metabolic Impairment in Acute and Chronic Cardiac Diseases

Several findings indicate that selective cardiac metabolic changes can occur in both acute and chronic cardiovascular perturbations. The metabolic impairment underlying the pathophysiology of coronary artery disease (CAD), HF and cardiovascular diseases (CVDs) are under continued investigation since the identification of specific metabolic targets could contribute to the development of potential therapeutic approaches.

### 3.1. Metabolic Changes Occurring during Acute Ischemia

According to the World Health Organization (WHO) Global Health Estimates, CAD is responsible for 16% of the world’s total deaths. In particular, myocardial infarction (MI) represents a leading cause of death in its acute phase even if its long-term consequences are also clinically relevant. The resulting tissue injury depends not only by ischemia *per se* and its duration, but also by oxygen restoration (reperfusion) through the use of thrombolytic therapy, primary percutaneous coronary intervention (PCI) or revascularization by coronary artery bypass graft surgery (CABG) [58,59]. Reperfusion can paradoxically induce progressive tissue damage, extending the necrosis and exacerbating the final harmful effects to the myocardium and coronary microcirculation. Therefore, both ischemia and reperfusion contribute to the final infarct size in an event known as “lethal reperfusion injury”, an irreversible injury characterized by apoptotic or necrotic tissue.

Metabolically, the acute cardiac ischemia is characterized by early modifications of substrates and energy metabolism variations derived from pH changes and reduced oxygen availability. The consequent mitochondrial metabolic dysfunction leads to a dramatic decrease in ATP formation by oxidative phosphorylation and to increased levels of intracellular inorganic phosphate [3,60,61]. During this condition, the ATP demand rapidly increases, while its relative production is not satisfactory, reflecting the augmented concentration of intracellular ADP; as adaptive response, the adenylate cyclase transforms ADP to ATP and AMP, a limited form of energy [3,60,61]. The elevation in AMP concentrations in turn activates the pro-survival AMP-activated protein kinase AMPK, which facilitates the glucose transport and glycolysis and fatty acid oxidation, representing a primary mechanism for conferring cardioprotection against reperfusion [62]. Indeed, this metabolic crossroads is crucial during the reperfusion process and could represent a potential metabolic therapeutic target.

During ischemia, the oxygen decline inevitably suppresses the metabolism of several macromolecules, including carbohydrates, fatty acids, amino acids and ketones. Therefore, the heart undergoes selective energetic changes to reduce the oxygen demand and maximize the substrate use. Initially there is a transfer of phosphate from phosphocreatine to ATP (via creatine kinase) for maximizing ATP preservation. However, this process becomes insufficient in the case of extensive ischemic hearts [63]. On the other hand, the heart tries to save further oxygen consumption by preferentially using glucose, a substrate that produces high-energy products with higher efficiency compared to fatty acid oxidation.

Therefore, the main energetic-metabolic modulation occurring during ischemia consists of shifting from aerobic to anaerobic energy production, activating the anaerobic glycolysis, stimulating the glucose myocardial uptake and inducing glycogen breakdown. The activation of the anaerobic metabolism by the heart is to be considered as an ischemia-response mechanism, whose aim is to ensure the ATP production necessary for cell survival and to preserve cell membrane integrity [63,64]. It is important to note that the glycolysis-dependent ATP can induce beneficial effect in a moderate ischemic heart due to its ability to control the ionic balance through the activities of the Na^+^/K^+^-ATPase pump in the sarcolemma and Ca^2+^ ATPase pump in the sarcoplasmic reticulum. However, in the severe ischemic heart, the persistent glycolysis can result in an intracellular pH decrease due to the increased proton (H^+^) production and lactate production that depress the myocardial contractile function, evincible after a few seconds or minutes of the ischemic event [65]. The excessive accumulation of H^+^ and lactate induces in turn the inhibition of glycolysis; therefore, fatty acid oxidation continues to be the predominant metabolic way also in the ischemic heart. As reported by several studies, the fatty acid oxidation is responsible for deleterious effects to the heart due to its inhibition of glucose oxidation, induction of further H^+^ and lactate production and alterations in the ion homeostasis, resulting in cardiac mechanical dysfunction [3,63,64]. Other studies have also demonstrated that acute ischemia can result in a hyper-efflux of potassium (K^+^), evinced by the typical changes in electrocardiographic repolarization that, together with alteration of Ca^2+^ concentration, can be responsible for arrhythmia and eventual cardiac arrest with a fatal outcome [66].

### 3.2. Cardiometabolism in Chronic Ischemic Heart Disease

When ischemia is able to induce myocardial necrosis with consequent loss of functional myocardium, there is no recovery of contractile function due to the progressive remodeling and fibrosis in the surrounding tissue. Conversely, if ischemia is not severe enough to induce necrosis, other responses (myocardial stunning, hibernation, preconditioning), that also depend by various factors particularly related to the duration and intensity of ischemia, as well as reperfusion and compensatory mechanisms (i.e., collateral circulation [67,68]), can be generated following a chronic ischemic event [69].

In the case of an intense and durable ischemia unable however to induce cell death, restoration of the blood flow generates a viable, but stunned myocardium presenting post-ischemic contractile dysfunction reversible after a period of days or weeks [70]. This can act as a physiological adaptive and protective response in order to minimize cellular and biochemical abnormalities induced by ischemia [71]. Accordingly, the post-ischemic transient reversible myocardial contractile dysfunction occurring in the stunned myocardium does not induce metabolic impairment.

The other possible outcome of myocardial ischemia derives from a chronic low blood flow that induces a metabolic heart adaptation for the maintaining of live cells. However, in this condition cells do not contract at rest and appear in a dedifferentiated state [70]. After the revascularization procedure, the “hibernating” cardiomyocytes can recover their function, even if the restoration time of the regional contractile function can be variable (occurring within days, months or up to a year), depending on the duration and intensity of the flow reduction and on the injury extension in the affected myocardial area. Indeed, in the involved myocardium it is possible to observe a partial or total recovery of cardiac function through the improvement of the coronary blood flow or the reduction of the myocardial oxygen demand [71].

The molecular mechanisms that are activated following both myocardial stunning and hibernation result in the induction of complex cytoprotective molecular processes and reorganization of gene and protein expression programs aiming to protect the chronically ischemic heart [71]. The metabolic adaptation of the hibernating myocardium is dictated by the coronary flow reduction; the relative response firstly consists in an auto-regulative coronary vasodilation for counteracting the pressure decline and in a decrease of contractility and energy demand to meet the imbalance between energy request of the hypo-perfused myocardium and supply, generating a myocardial state of “perfusion-contraction matching” [72]. The functional and metabolic down-regulation of hibernating myocardium also depends on the typical cytostructural alterations, where a reduction in phosphates and a shift from oxidative to anaerobic metabolism occurs. In particular, a persistent stunning inducing myocardial hibernation shifts the utilization of substrate from fatty acids to glucose, increasing the glycogen depots through a decreased activity of GSK-3β, responsible for the glycogen synthase inactivation [73,74]. The GSK-3β downregulation represents a well-known key element for inducing the metabolic adaptation of hibernating myocardium and for preserving its left ventricular function during subsequent episodes of acute ischemia [73]. The other possible myocardial response to ischemia is represented by ischemic preconditioning (IPC), a phenomenon based on a brief and repetitive cycles of sub-lethal ischemia and reperfusion, subsequent to an imbalance between myocardial metabolic request and myocardial oxygen supply, which cannot be satisfactory due to the presence of CAD and limited coronary reserve. Complex adaptive processes, mostly converging on the modulation of the mitochondrial PTP (permeability transition pore), can be activated as a self-protective response of the myocardium against further ischemia events [59].

### 3.3. Metabolic Changes Occurring during Reperfusion

As mentioned above, blood flow by reopening the occluded coronary artery represents the most effective therapeutic intervention for the ischemic myocardium. Indeed, without reperfusion, the dramatic decrease of ATP availability and the high Ca2+ levels induce myocyte atrophy and cell death [59]. However, this phenomenon can reduce the beneficial effects of myocardial reperfusion *per se* since it is associated with a complex array of intracellular damaging events culminating in cardiomyocyte death by necrosis or apoptosis [59]. It is known that oxidative/nitrosative stress generated by reperfusion is a major cause for myocardial damage consequent to the harmful action exerted by reactive oxygen species (ROS) and peroxynitrite (ONOO–) on myocardial fibers [69,75].

In addition, the excessive calcium influx dependent by the activation of the Na^+^/Ca^2+^ exchanger, deriving from a sequence of specific ionic pump alterations, ultimately induces cellular and mitochondrial calcium overload, and myofibril hypercontracture (as evinced by diastolic ventricular pressure increase) [76]. The loss of ionic homeostasis alters the permeability of the inner mitochondrial membrane and the intracellular Ca^2+^ overload, associated with ROS generation occurring during early reperfusion. This leads to the opening of the mitochondrial PTP that uncouples oxidative phosphorylation resulting in cell death [77].

The tissue damage dependent on an ischemic event is exacerbated by an hyperinflammatory response; this is generated by cytokines and cell-adhesion molecules expressed by parenchymal and endothelial cells that induce the recruitment of circulating neutrophils to the re-perfused zone, and by cytosolic components released by necrotic cells [59,78]. Neutrophils in turn induce vascular plugging and degradative enzymes and further ROS release [59,78].

Among the several events that mediate the reperfusion-dependent myocardial dysfunction, the metabolic modulation represents a fundamental element that participates in the pathogenesis of the acutely ischemic heart.

Particularly, following a brief period of ischemia, several metabolic impairments characterize the heart at the onset of reperfusion even if these alterations are, almost always, unable to culminate in cell death. This condition, as part of the myocardial stunning, is associated with reversible modifications in terms of ATP depletion, cellular and mitochondrial swelling, alteration of microvascular permeability and endothelial dysfunction [79]. However, in the case of prolonged ischemia, irreversible damage occurs during reperfusion; the levels of lactate, ions and creatine phosphate become dramatically increased, the Ca^2+^ concentrations augment and severe mitochondrial damage can be observed. The irreversible impairment of the cardiomyocyte membrane leads to cell necrosis and apoptosis.

If the ischemic heart is exposed to a prompt reperfusion, oxygen delivery to the heart can recover the mitochondrial oxidative phosphorylation, glucose and fatty acid oxidation tend to normalize, although at a different rate. In fact, in the initial phase of the reperfusion anaerobic glycolysis rates remain elevated and the high circulating levels of fatty acids, and the consequent alteration of their oxidation occurring during ischemia, allow a quicker recovery of fatty acid oxidation [60,64]. Therefore, the competition between the fatty acids and glucose oxidation is in favor of fatty acid, whose circulating levels diminish the recovery of glucose oxidation through the glucose fatty-acid cycle or ‘Randle cycle’ [64,80]. In this regard, diverse studies demonstrated that the high circulating levels of fatty acids, detected during myocardial ischemia due the activation of the peripheral sympathetic nervous system, can significantly participate to the extension of cardiac injury [81].

ATP recovery secondary to blood flow restoration facilitates lactic acid export, with a trend of the intracellular pH toward normalization. However, the uncoupling of glycolysis and glucose oxidation leads to the production of both protons and lactate in the reperfusion phase. It should also be noted that the continuous elevation of intracellular Ca^2+^ concentration secondary due to a damaged sarcolemmal membrane, as well as the increased Ca^2+^ entry into the mitochondria dictated by the restoration of the mitochondrial membrane potential, can also activate the mitochondrial PTP [82]. Accordingly, if during ischemia, mitochondria are able to control the abnormal intracellular Ca^2+^ levels preserving their capacity to transport it, reperfusion leads to mitochondrial structural alterations. Therefore, Ca^2+^ transport becomes excessive and great amounts of energy are consumed [83]. Thus, cardiomyocytes undergo apoptosis, even if this cell death type is less frequent than necrosis in I/R damage; both events induce release of cytokines, chemokines and other proinflammatory factors, recruiting neutrophils that infiltrate the ischemic tissue, exacerbating the final damage [84].

### 3.4. Cardiometabolic Profile in Inherited Cardiomyopathies

Among the complexity of heart failure (HF) phenotype, inherited factors can also be causative. Inherited cardiomyopathies show selective myocardial metabolic changes that correlate with HF progression and severity. The most inherited cardiomyopathies caused by autosomal dominant transmission of single-gene disorders include hypertrophic cardiomyopathy, dilated cardiomyopathy and arrhythmogenic cardiomyopathy [85] (Table 1).

#### 3.4.1. Fatty Acid Oxidation Disorders (FAODs)

In the context of hereditary cardiomyopathies, fatty acid oxidation disorders (FAODs) are categorized as metabolic abnormalities involving both β-oxidation and fatty acid transport into the mitochondria, through the carnitine system [119]. Several studies indicate that mutations in SLC22A5, SLC25A20, CAC genes can cause primary carnitine deficiencies (PCD) [86,120,121]. In this regard, defects in carnitine transporter (CTD, 5q mutation), carnitine-palmitoyl transferase 1 and 2 deficiencies (CPT-1 and CPT-2), the lack of carnitine acylcarnitine translocate (CACT, 3p21 mutation), have been linked to cardio-metabolic disorders that usually determine the onset of cardiomyopathy, arrhythmia, conduction disease and HF [107,122].

On the other hand, mutations in enzyme-coding genes involved in β-oxidation process, and particularly very-long-chain acyl-CoA dehydrogenase (VLCAD) and long-chain 3-hydroxy-acyl-CoA dehydrogenase (LCHAD), have also been associated with impaired fat metabolism, cardiac lipidosis and reduced ketone body production [86,107,123].

#### 3.4.2. Glycogen Storage Diseases (GSDs)

Glycogen storage diseases (GSDs) are known to be caused by inherited defects of key enzymes involved in synthesis, breakdown or accumulation of glycogen [124]. To date, approximately 14 types of glycogen storage disorders have been characterized; among these, GSD types II, III, IV and VI are recognized to target the cardiac metabolism causing restrictive or dilated cardiomyopathies, left ventricular hypertrophy and conduction disease. Few cases have been reported in literature in which phosphorylase kinase deficiency (GSD IX) and the lack of glycogen synthase enzyme (a clinical condition usually known as GSD 0) have caused hypertrophic cardiomyopathy and sudden cardiac arrest [107,108,125]. Several pieces of evidence have demonstrated that mutations in the GAA gene localized on chromosome 17 (Pompe disease, GSD II), coding for acid α-glucosidase as well as defects in the LAMP2 gene (Danon disease, GSD IIb), are dramatically involved in the alterations of cardiac metabolism; this in turn induces glycogen accumulation, altered autophagy and abnormalities in the expression of mitochondrial genes, with consequent alteration of mitochondrial respiration [126,127,128,129,130]. Likewise, mutations in AGL gene (Cori disease, GSD III) provoke amylo-1,6-glucosidase deficiency and abnormalities in glycogen metabolism [107].

PRKAG2 cardiac syndrome belongs to GSDs and it is characterized by mutations determining abnormal activity of AMPK, glycogen deposits and activation of mechanistic target of rapamycin (mTOR) pathway, showing clinical features of hypertrophy and conduction disease [131,132,133,134,135].

#### 3.4.3. Lysosomal Storage Disorders (LSDs)

Lysosomal storage disorders (LSDs) represent a particular class of metabolic abnormalities characterized by deficit in the enzymes present in the lysosomal compartment, that determine the accumulation of partially degraded macromolecules. Hereditary pathological conditions, such as sphingolipidosis, mucopolysaccharidosis, mucolipidosis, belong to this class of abnormalities [107].

Studies indicate that mutations in selective genes, such as GLA (Xq22 region, encoding for α-galactosidase A), GBA1 (1q21 region, coding for glucocerebrosidase), SMPD1 (11p15 region, coding for acid sphingomyelinase), GLB1 (3p22 region, encoding for β-galactosidase) as well as mutations of 11 genes (IDUA, IDS, SGSH, NAGLU, HGSNAT, GNS, GALNS, GLB1, ARSB, GUSB, HYAL1) encoding for enzymes involved in the degradation of glycosaminoglycans (GAGs), may negatively affect the substrate catabolism. Consequently, it appears that the accumulation of glycosphingolipids such as globotriaosylceramide, glucosylceramide, sphingomyelin, GM1 ganglioside and GAGs that can lead to decreased activity of respiratory chain enzymes, release of proinflammatory cytokines, growth factors and oxidative stress. Taken together, these events can worsen heart function, leading to hypertrophy and conduction abnormalities [87,107,109,110,112,114,116,117].

#### 3.4.4. Mitochondrial Disorders

Mitochondrial disorders may derive by mutations transferred by matrilineal inheritance or through sporadic mutations affecting mitochondrial or nuclear DNA with remarkable cardiac involvement. Several studies highlighted that mutations in protein-encoding genes participating to the oxidative phosphorylation and/or coded for respiratory chain complexes (ACAD9, NDUFAF1, SDHA, SDHD, MTCYB, COX6B1, MT-CO1/2/3, COA5, COX10, SCO1/2, SURF1, ATP6, ATP8, TMEM70, CQQ2, CQQ4, CQQ9) have been associated with alterations of electron transport chain and reduced energy production into mitochondria. Similarly, genetic changes in FXN (causing GAA triplet expansion disease, also known as Friedreich’s ataxia) and TAZ gene mutations have been associated with oxidative stress, mitochondrial dysfunction and impaired iron metabolism correlating with cardiac hypertrophy or dilated cardiomyopathies [86,107] and references therein.

## 4. Cardiometabolic Adaptations in Heart Failure and Chronic Cardiac Diseases

It is extensively reported that several events, including ischemic insult, MI, hypertrophy and pressure overload, might be responsible for the cardiac pathological remodeling in terms of both contractile and metabolic functions [3,136]. Table 2 recapitulates the main cardiometabolic alterations occurring in both acute and chronic cardiac diseases. It is also widely accepted that chronic metabolic alterations secondary to obesity, insulin resistance, dyslipidemia and type 2 diabetes mellitus (T2DM) represent important risk factors for CVDs, including CAD and HF [137]. Through a complex spectrum of metabolic interactions, these main cardiovascular risk factors can induce both direct adverse actions to the myocardium and indirect effects to the vascular system.

Despite the advancement in medical therapy for the treatment of chronic HF, this syndrome continues to have an enormous impact (64.3 million people are suffering from HF worldwide with a prevalence of 1–2% in the general adult population of developed countries [149,150]) and is still associated with a significant increase in the total annual cases [149,150]. Chronic HF is a complex and multifactorial disease, where several co-existing causative factors can lead to the syndrome and various comorbidity conditions increasing the severity of HF, considered as the chronic stage of any disease leading to cardiac functional impairment, as well as the final clinical event of CVDs [150,151].

From an energetic viewpoint, the energetic impairment and the flexibility of the heart to metabolically adapt to the pathological changes occurring in a failing heart play a crucial function in the pathogenesis of HF; accordingly, several approaches targeting the modulation of cardiac metabolism achieved promising results for the treatment of HF over the past years. These relatively recent findings have represented the concretization of numerous studies on myocardial energetic from the 1940s and are still alimented by a considerable number of studies aiming to identify further metabolic targets to be exploited in clinic.

The typical metabolic impairment occurring in HF concerns changes in substrate utilization, oxidative phosphorylation and high-energy phosphate metabolism. The shift from fatty acid to glucose utilization for obtaining energy is accompanied by a decreased oxidative metabolism and energy reserve [152]. These events induce mitochondrial dysfunction and ATP deficiency that ultimately alter contractile function [88,153] (Table 2).

It is known that if the predominant cardiac metabolism is based on carbohydrate use for obtaining energy before birth, in the post-natal phase, the oxidation of fatty acids becomes the main source of energy and linearly correlates with a molecular and genetic profile typical of the adult phenotype [154,155]. However, several pathophysiological conditions (as indicated above) reflect specific cardiometabolic profiles mainly based on the overturning of the energy substrate utilization from fatty acid oxidation to glucose oxidation and on the reprogramming of a selective fetal gene profile, including the expression of atrial natriuretic factor (ANF), transforming growth factor β (TGF-β) and early response genes, such as c-myc and c-fos [154]. This cardiac metabolic remodeling based on the reversion to fetal metabolism is particularly evident in the HF secondary to pathological cardiac hypertrophy.

### 4.1. The Shift towards Glucose Utilization in the Failing Heart

The increase in glucose uptake, through GLUT1 or GLUT4, and glycolysis rate are common events of hypertrophied and failing hearts. It has been demonstrated that the increased glucose entry into the cell and the activation of the rate-limiting enzyme phosphofructokinase lead to an augmented flux through the glycolytic pathway in the heart with pressure-overload left ventricular hypertrophy [156]. The improved glucose entry could represent an important mechanism to induce glucose uptake, necessary to maintain a normal cardiac function; it is presumable that this metabolic mechanism represents a compensatory response occurring in HF for improving the oxygen efficiency for ATP production in a condition in which oxygen availability becomes limited [153].

In this regard, some data appeared discordant and the possibility that the shift towards glucose utilization in HF could be considered a putative maladaptive response, as well as an integral pathological process, represented an intensely debated issue.

#### 4.1.1. Is the Induction of Glucose Utilization a Double-Edged Sword in Heart Failure?

Important findings showed that the increased glucose utilization represents a compensatory mechanism to the down-regulated fatty acid metabolism occurring during hypertrophy. Important information about this has also been provided by works carried out using genetic and transgenic mouse models, as well as isolated working heart preparations. In particular, several genetic model-based studies showed that the decrease in glucose utilization appears deleterious for cardiac failure and hypertrophy, while its use in hypertrophied hearts of mice overexpressing GLUT1 exerts protective effect against contractile dysfunction and cardiac dilation after chronic pressure overload [157]. This suggests that increasing the availability of cardiac glucose could regulate the hypertrophic program, thus exerting beneficial effects against myocardial dysfunction. Other studies demonstrated that also the cardiac deficiency of GLUT4 (the main glucose receptor present in the adult heart and the most important transporter responsible for the cardiac glucose uptake [158]) induces hypertrophy and profound disturbances in Ca^2+^ and pH homoeostasis in a rodent model of insulin-resistant cardiomyopathy [159]. These findings provide important information about the potential therapeutic significance to target GLUT4 in the diabetic cardiomyopathy and suggest that the loss of insulin-dependent glucose uptake can induce pathological responses [159]. Similar results have been obtained on the isolated working heart preparation, a widely used experimental model that is ideal to simultaneously record different parameters of cardiac function and energy substrate metabolism, in particular related to glucose and fatty acids metabolism in both physiological and pathological conditions.

In particular, by exposing the hearts of mice with cardiac-selective ablation of the GLUT4 gene to global low-flow ischemia, Tian and Abel [160] demonstrated that the increased glucose uptake occurring during ischemia is crucial for protecting the heart and for the myocardial post-ischemic recovery. These evidences further support the hypothesis that GLUT4-dependent glucose transport mediates crucial cardioprotection against ischemic insult. Moreover, Wambolt et al. [161] observed higher rates of glycolysis from exogenous glucose and glycogen turnover in hypertrophic hearts compared to control hearts exposed to severe ischemia, suggesting that hypertrophy induces significant differences in the metabolism of exogenous glucose and glycogen.

Other mechanistic evidences in small pre-clinical models indicate that also increasing the glucose utilization by overexpressing hexokinase-2, the enzyme that phosphorylates glucose to form glucose-6-phosphate that represents the initial phase of glucose metabolism, is a crucial beneficial actor against cardiac hypertrophy in response to chronic β-adrenergic stimulation [162,163]. These results suggest that glucose phosphorylation is an important step determining cardiac glucose utilization, as also demonstrated by Liang and colleagues [164], according to whom the increase of the hexokinase activity resulted in enhanced glycolysis and increased glycogen storage in the heart [164]. Notably, glucose phosphorylation by hexokinase is the initiating step in all the pathways using glucose, such as glycolysis and glycogenesis in addition to the pentose phosphate and hexosamine biosynthetic pathways [165]; data demonstrated that these last two processes also resulted enhanced in models of hypertrophy [166,167].

The hypothesis that increased glucose utilization is compensatory during cardiac hypertrophy derive from studies on mice models with myocardium-specific overexpression of PPARα, a nuclear receptor that transcriptionally controls the cardiac energy metabolism and that is strongly implicated in the hepatic metabolic response to diabetes mellitus [168,169]. Here, mice exhibiting increased myocardial fatty acid oxidation rates and decreased glucose uptake and oxidation through the cardiac-restricted overexpression of PPARα resembled the phenotype of diabetic cardiomyopathy, including ventricular hypertrophy [170]. On the other hand, the ablation of PGC-1α gene, a master regulator of oxidative metabolism and mitochondrial function in several tissues, in particular in the heart, induces energetic cardiac defects resulting in cardiac dysfunction, in addition to hypertrophy and HF progression after pressure overload [171,172].

Notwithstanding these reports, it should be considered that specific conditions characterized by obligate cardiac use of glucose can correlate with hypertrophy development. This is the case of some studies on isolated hearts exposed to high workload in the presence of glucose, indicating that glucose can induce a metabolic remodeling preceding and inducing structural and functional cardiac dysfunction [173]. Other reports showed that the increased myocardial glucose uptake precedes the development of left ventricular hypertrophy in hypertensive humans [174]. Furthermore, patients treated with a tyrosine kinase inhibitor (sunitinib), able to increase cardiac glucose uptake, exhibit activation of the fetal gene program developing cardiac dysfunction or failure [175,176]. These results suggest that reliance on glucose can be harmful for the heart [177].

Moreover, it has been demonstrated that although the short-term cardiac-specific induction of GLUT1 at the onset of pressure overload hypertrophy induced in mice can mitigate the altered structural and mitochondrial remodeling, it is unable to maintain contractile function [178]. These findings provide evidence that excess glucose might induce potential short-term harmful action that impairs the cardiac function. Other evidence supporting this hypothesis was achieved by Yan et al., using transgenic mice with cardiac-specific overexpression of GLUT1 [179]; the authors demonstrated how augmented rates of myocardial glucose uptake and oxidation can predispose the heart to impaired function. To further address the mechanism by which glucose might alter the cardiac function, Wende et al. [180] investigated the consequences to restore glucose delivery in a context of short-term diabetes onset using transgenic mice with inducible cardiac-specific expression of GLUT4. This study showed that the increased myocardial glucose delivery associates with an accelerated mitochondrial dysfunction in diabetic cardiomyopathy, indicating how reducing the glucose uptake during uncontrolled hyperglycemic conditions could represent an important therapeutic intervention to limit glucotoxicity.

#### 4.1.2. Significance of Glucose Utilization during Heart Failure

On the whole, it is still controversial whether the metabolic changes are the basis of an adaptive or maladaptive response during cardiac hypertrophy and HF and other concerns remain to be clarified. Based on the available data, it is possible to claim that the increased glucose metabolic utilization induces adaptive response as long as the energetic demand is met in the normal heart. However, the protracted metabolic remodeling related to the hyper-reliance of glucose could alter the adaptive capacity of the heart to the substrate’s utilization contributing to disease progression [152].

During HF the metabolic rearrangements induce a progressive decrease of cardiac energy production generated by a continuous impairment of substrate use and mitochondrial function. In particular, the metabolic changes are due to alterations in substrate flux rather than to the absence of substrate availability, generating a metabolic imbalance that negatively affects the cardiac function, culminating in a failing heart which can be considered as an “engine out of fuel” [59]. The progressive increase of the metabolic demand induced by the continued activation of the sympathetic nervous system typical of HF can worsen the cardiac dysfunction [181]. In evaluating the metabolic changes in HF and chronic-related cardiac pathological states, it is therefore important to consider the influence of several additional factors, including the degrees of workload and wall stress, as well as concomitant cardiovascular risk factors. Furthermore, the metabolic profile differs between different contexts inducing HF and myocardial adaptation is also influenced by the different clinical stages characterizing the progression of HF (i.e., compensated hypertrophy with or without diastolic dysfunction versus manifest systolic dysfunction) [153].

### 4.2. Impact of Glycogen Metabolism during Heart Failure

Among the myocardial energetic changes occurring during HF, the glycogen metabolism and its controlled breakdown should not be underestimated, also considering that glucose 6-phosphate is a substrate for glycogen synthesis, as well.

As evidenced in the fetal heart, the failing heart also presents high levels of glycogen, which appears essential for the heart’s development, as demonstrated by the perinatal death of mice presenting disrupted isoform 1 of glycogen synthase caused by severe cardiac defects and heart dysfunction [182]. The shunting of intracellular free glucose into glycogen protects cellular proteins from excess glycosylation, thus mitigating the glucotoxicity [183] and several studies indicate a positive correlation between cardioprotection against ischemic injury and other cardiac pathologies and glycogen availability [184]. Furthermore, myocardial glycogen stores represent a crucial source of glucose to support the cardiac function not only in the normal heart, but also in the hypertrophied heart [185]. The contribution of glycogen metabolism in the hypertrophied heart during severe ischemia is of particular importance since rates of glycolysis from both exogenous glucose and glycogen result in being augmented in the hypertrophied heart, along with the increase in glycogen turnover [185], suggesting that glycogen homeostasis and metabolism represent important metabolic targets with potential therapeutic impact during HF.

### 4.3. Role of Free Fatty Acid Metabolism in the Failing Heart

During HF, alterations in transcription of key enzymes involved in fatty acid metabolism are detected [186]. Since HF can correlate with increased circulating fatty acid levels due to high lipolysis rates, several studies in both pre-clinical models and patients with cardiac hypertrophy and HF demonstrated the beneficial action of selective inhibitors of key steps of fatty acid oxidation. [187]. On the contrary, other studies reported decreased myocardial fatty acid oxidation rates in the failing heart, as evinced by decreased transcriptional level of genes involved in fatty acid oxidation, making the impact of fatty acid oxidation a complex issue in the context of HF. However, on examining the myocardial energetic profiles during HF progression, it can be observed that the increased glucose utilization mainly occurs during the early stage of the syndrome, when the metabolic shift in substrate utilization is still not significantly evident (i.e., fatty acid use is still unchanged) [186]. Conversely, in the advanced stage of HF, the fatty acid and glucose utilization appear decreased, the latter due to the myocardial insulin resistance development [186], with consequent alteration in the energy transfer to the myofibrils. Notably, the contribution played by circulating free fatty acids, glucose and insulin that increase in HF should be also considered for discerning the involvement of intrinsic metabolic adaptation related to fatty acid and glucose metabolism during HF [186]. Accordingly, myocardial insulin resistance inevitably leads to altered intracellular insulin signaling with consequent reduced cardiac responsiveness to the physiological circulating levels of insulin and decreased insulin stimulation of glucose uptake and oxidation [188].

Indeed, this condition, which is predominant in obesity and T2DM, is also characterized by elevations in circulating free fatty acids; the resulting imbalance between fatty acid uptake and mitochondrial β-oxidation induces fatty acid accumulation and lipotoxicity driving the genesis of myocardial insulin resistance that culminates in myocardial mechanical dysfunction [188]. Alterations in fatty acid β-oxidation also represent a significant contributor to the development of cardiac dysfunction in HF and ischemic heart disease, thus its inhibition can represent an important metabolic target for the treatment of cardiac function deficiency associated with dysmetabolic states and ischemia [63]. In support of this, many studies reported that a specific “metabolic footprint” occurs in DMT2, obesity and metabolic syndrome, as recently reviewed by Gibb and Hill [3]. Here, the authors greatly highlighted how, in addition to lower rates of glucose oxidation, glycolysis and lactate oxidation, main triggers of left ventricular hypertrophy and diastolic dysfunction in the diabetic heart are represented by higher rates of fatty acid and ketone oxidation. The resulting glucotoxicity and lipotoxicity in turn increase the amount of advanced glycation end products and ROS that induce mitochondrial dysfunction and cardiac insulin resistance [3].

### 4.4. The Red Skeletal Muscle as a Counterpart: Common and Different Metabolic Traits

Numerous studies have comparatively investigated the metabolic profile of the myocardium and the skeletal muscle under normal and physiopathological conditions, emphasizing the common and different traits of these striated red muscle tissues. In contrast to the skeletal muscle, the myocardium must beat continuously and rhythmically in order to maintain its structural and functional integrity. The diverse metabolic properties between the cardiac and the red skeletal muscle strictly depend on their different morphological structure and molecular setting [189,190,191]. In contrast to the cardiac muscle, which is flexible in the choice of energy substrates and shows a relatively low dependency on glucose and glycogen, the red aerobic skeletal muscle selects the substrates on the basis of the duration and intensity of the exercise. Indeed, under resting, fasting and low intensity exercise, skeletal muscle mainly draws energy from fatty acids and their oxidation. The increased β-adrenergic stimulation, detectable in low- and moderate-intensity exercise, induces lipolysis in the adipose tissue generating free fatty acids which are used to support the muscle activity. However, the rate of fatty acid oxidation decreases under high intensity exercise in favor of carbohydrate metabolism [192]. Glucose is crucial for contraction under prolonged exercise. Training increases glucose uptake, glycolysis, glucose oxidation and glycogenesis due to the activation of AMPK and the translocation of GLUT4 [193]. Of note, skeletal red muscle metabolism undergoes adaptations which are related not only to exercise intensity and duration but also to muscle size, fiber distribution and rate of contraction [194].

As in the myocardium, ketone bodies represent an alternative energy source for the skeletal muscle, mainly contributing under prolonged fasting, exercise, excess of fatty acid availability and low carbohydrate consumption [195].

Under cardiac pathologies, such as HF, typical alterations of skeletal muscle morphology, metabolism and function are observed. These abnormalities depend on different factors, including inadequate oxygen delivery and excessive exposure to neurohumoral stimuli [196,197]. Under these conditions, the skeletal muscle biochemical profile is characterized by phosphocreatine and fatty acid metabolism changes. In particular, the impaired fatty acid catabolism reduces ATP production and mitochondrial function with consequent accumulation of lipid and limited exercise tolerance [196,197,198].

## 5. Therapeutic Strategies in Cardiac Diseases

Starting from the assumption that under pathological conditions, the shift of the heart to a glucose metabolism is an adaptive/protective stratagem [199], the promotion of carbohydrate utilization may represent a useful therapeutic strategy. Nonetheless, as previously mentioned, the presence of uncontrolled hyperglycemia may worsen cardiac function by glucotoxicity in diabetic patients [180].

In general, the goal of each anti-ischemic and cardioprotective pharmacological approach is based on its ability to reduce significantly the I/R-dependent myocardial injury. Interventions aiming to attenuate the detrimental effects of metabolic alterations secondary to acute I/R injury could protect the myocardium by reducing the infarct size, preserving the left ventricular function and preventing the onset of HF.

### 5.1. Metabolic Therapies in Heart Failure

During the past years, since HF is known to associate with mitochondrial dysfunction as a consequence of oxidative stress, antioxidants have been tested as possible protective strategies [200]. However, the complexity of this severe condition prompted researchers to consider new therapeutic frontiers.

Several attempts have been made to reduce the upstream fatty acid supply to the heart, i.e., reducing their plasma levels, but without encouraging results. It is the case of the lipolysis inhibitor acipimox, that was able to increase glucose utilization by the heart, without inducing an improvement of cardiac function [201,202]. These results demonstrated that the restriction of the available fatty acids does not represent the right way to achieve the pursued goal. For this reason, more attention was paid to downstream enzymes, such as CPT-I. In particular, the inhibition of this enzyme (by etomoxir, perhexiline, and oxfenicine) reduces fatty acid oxidation, thus increasing the use of glucose, rather than decrease their plasma levels [2]. More specifically, experimental evidence demonstrated that etomoxir improves the cardiac performance through the sarcoendoplasmic reticulum calcium ATPase [203,204]. In general, the inhibition of CPT-I resulted in being associated with a significant recovery of cardiac function not only in animal models but also in human trials [205,206,207,208].

Many experimental data point towards dichloroacetate (DCA), a pyruvate kinase dehydrogenate (PDK) inhibitor, as a possible cardioprotective tool via increasing the activity of pyruvate dehydrogenase (PDH), promoting glucose oxidation [209,210,211,212]. However, even if promising, the chronic neurotoxicity of DCA limited its application in humans [213,214]. Another possibility to limit fatty acid oxidation is represented by the activation of malonyl CoA decarboxylase since it may inhibit the translocation of fatty acids into the mitochondria. This strategy reduces fatty acid oxidation and confers cardioprotection after ischemic events in animal models, but a role in HF still remains to be fully elucidated [5].

From another point of view, targeting insulin sensitivity could be of interest, given that insulin resistance is one of the principle independent causes of HF [215]. Indeed, oral hypoglycemic and insulin sensitizing agents such as thiazolidinediones, acting as PPARγ agonists, ameliorate glucose utilization also improving cardiac performance after ischemia [216,217].

In the context of metabolic diseases, such as T2DM, studies aimed to prove cardiovascular safety of antidiabetic therapies, described the sodium–glucose cotransporter 2 (SGLT2) inhibitors as able to reduce the incidence of HF [218]. Since the human heart does not express SGLT2, the beneficial effects of SGLT2 inhibitors on the cardiac muscle are supposed to be indirect and linked to their systemic action [219]. Even if the exact mechanism operating on the heart is still unknown, data from animal models depict a role for the myocardial Na^+^/H^+^ exchanger and for oxidative stress. Indeed, SGLT2 inhibitors could reduce the activity of Na^+^/H^+^ exchanger improving the excitation–contraction coupling in cardiomyocytes and increasing the antioxidant ability of mitochondria [220,221,222]. Despite the lack of evidence regarding the specific machinery responsible for SGLT2 cardiac beneficial effects, important clinical trials strongly support their positive outcomes on HF [223,224,225,226,227].

In addition, data from both experimental and clinical trials describe metformin, one of the most used therapies in diabetes, as a protective agent not only in T2DM but also with regard to HF. Metformin is supposed to act by reducing oxidative stress and inflammation, and by improving endothelial function [228,229,230,231,232,233,234]. It is likely that, operating as an AMPK activator, metformin is able to raise glucose uptake in cardiomyocytes inducing a general improvement of cardiac structure and function [235,236,237].

In the field of CVD associated with metabolic pathologies, promising results have been reported for glucagon-like peptide-1 (GLP-1), an endogenous insulin secretagogue that increases glucose utilization in the heart, globally improving cardiac function after acute myocardial infarction [238]. Today, it is known that Glucagon-like peptide-1 receptor analogs (GLP-1RAs) not only improve glycemic control but also reduce body weight and blood pressure [239]. In particular, a significant reduction in the risk of developing CVD events, such as HF, was observed in a large-scale trial testing the GLP-1RA Liraglutide (Liraglutide Effect and Action in Diabetes: Evaluation of Cardiovascular Outcome Results, LEADER trial) [240]. Accordingly, two more clinical trials, SUSTAIN-6 [241] and REWIND [242] showed a decrease in the rates of major adverse cardiovascular events (Table 3).

It still remains to be elucidated whether the beneficial effects of anti-diabetic drugs on CVD outcomes only reflect the positive systemic actions elicited by these molecules.

### 5.2. Metabolic Therapies in Myocardial Infarction

The possibility to modulate the metabolic changes that are related to I/R injury may offer an important opportunity in the development of clinical treatments. As previously described, during an ischemic event several factors contribute to an increase of glycolysis [136].

It is known that during/after ischemia, the heart is protected as long as glycolysis goes on, demonstrating that it is the ATP derived from this pathway to confer cardioprotection [243]. Indeed, the ischemia-dependent cardiac contracture starts when glycolysis is interrupted, causing a reduction of ATP that impairs the activity of critical enzymes [261]. In this context, in animal models of ischemia, cardioprotection has been achieved by increasing glucose and insulin levels, or by raising the use of endogenous glycogen [262,263]. Promising results, in this respect, came from the use of AMPK activators, able to improve cardiac performance recovery and to reduce the infarct size [264,265]. In fact, AMPK is a key component of the well-established cardioprotective IPC which, surprisingly, is strongly impaired in the absence of glucose, suggesting the importance of aerobic glycolysis in the early reperfusion [266]. The importance of increasing the glycolytic pathway during/after an ischemic event to protect the cardiac muscle is strongly emerging, making this metabolic path a hopeful therapeutic target.

Glucose is also used by the HBP, but to date the role of this pathway in cardioprotection is still controversial and debated [68]. More likely, a possible target to prevent ischemia-induced injury may be the malate-aspartate shuttle (MAS), whose transient inhibition before or after ischemia (for example by aminooxyacetate) demonstrated protective effects on the heart [267,268,269]. Oxidative stress is a pivotal actor under ischemic insults; in this context, MAS inhibition is able to decrease the production of ROS induced by succinate, reducing the oxidative stress visible during the reperfusion and responsible for cardiomyocyte injury [270].

Looking at ischemia, another important feature that characterizes this condition is the significative reduction of glucose oxidation, whose reactivation depends on the availability of fatty acids that compete with glucose to enter this pathway [68]. So, stimulation of glucose oxidation is supposed to be a possible beneficial mechanism in the presence of myocardial infarction because of its ability to improve oxygen utilization [244] and to reduce proton overload [245]. The importance of glucose oxidation in cardioprotection is also supported by data showing that under IPC maneuver the pathway is strongly activated [246,269].

On this basis, the possibility to modulate the Randle cycle may represent a useful therapeutic mechanism in promoting glucose utilization and reducing fatty acid metabolism, with significative cardioprotective effects. These may depend on a better metabolic efficiency of the heart, since glucose oxidation, compared to FA, uses less oxygen to produce the same amount of ATP [247]. Indeed, the Randle cycle describes the reciprocal relationship existing between glucose and FA oxidation, i.e., a dynamic adaptation of the cells concerning the availability of energetic substrates [80,271]. Experimental evidences demonstrated that during the early reperfusion, AMPK activation increases fatty acid oxidation [248]; that in turn reduces glucose oxidation modulating the Randle cycle [248,249]. However, this event can be reverted by PDH activation when reperfusion induces a Ca^2+^ overload [250]. The Randle cycle can also be switched, in favor of glucose, indirectly by inhibiting fatty acid oxidation and/or their uptake [251,252].

In this regard, trimetazidine, an inhibitor of FA oxidation, is emerging as a medically accepted strategy for its ability to shift cardiac metabolism toward the use of glucose [253]. Indeed, in animal models of right ventricle hypertrophy and failure, the inhibition of FA oxidation by trimetazidine enhances glucose oxidation ameliorating the cardiac function [254]. Trimetazidine beneficial effects in ischemia and HF are mainly due to its ability to act as a competitive inhibitor of long-chain 3-ketoacyl CoA thiolase (3-KAT), the last enzyme of FA oxidation [255]. This inhibition reverses the Randle cycle increasing glucose oxidation with positive cardiac effects [256]. Clinical data demonstrated that trimetazidine administration in HF-affected patients is able to ameliorate the symptoms, improving cardiac function and clinical outcomes [257]. As in the case of HF, several ex vivo studies indicated that DCA is able to ameliorate cardiac performance recovery also under ischemia [258,259,260,272]. In vivo experiments highlighted the protective effects of acute administration of not only DCA [273] but also of reconstituted HDL (high-density lipoproteins) [274] and of phosphate compounds [275]. Similarly to HF, GLP-1ARs were able to improve glucose oxidation and promote cardiac recovery also in I/R events and in the presence of metabolic disorders [276,277].

Recently, glucose, insulin and potassium (GIK) infusion, firstly proposed by Sodi-Pallares and collaborators [278] has been accepted as able to induce a shift to glucose metabolism, protecting the heart during infarction. Experimental data obtained on animal models showed that treatment with GIK after an ischemic event may promote glycolysis, reducing cardiac injury and improving cardiac performance [279,280,281]. Clinical data from the trial named IMMEDIATE (Immediate Myocardial Metabolic Enhancement During Initial Assessment and Treatment in Emergency care) further supported the protective ability of GIK, due to the promotion of glucose metabolism instead of the fatty acid use [249]. Resulting findings confirmed an improvement of clinical outcomes [282,283]. Moreover, a randomized controlled trial on patients undergoing cardiopulmonary bypass found that GIK administration contributes to preserve cardiac muscle function [284] (Table 3).

Another important factor to consider within cardiac metabolism is the F0F1-ATP/synthase. Indeed, under hypoxic conditions, this crucial enzyme starts to dissipate energy, hydrolyzing ATP to extrude H^+^ [74,285,286], increasing ROS production, and causing a raise of mPTP that contribute to cell death [68,286,287]. Therapeutic intervention on F0F1-ATP/synthase may positively impact mPTP activity, recognized as crucial regulators of cardiomyocyte survival, but further investigations in the field are still lacking.

The possibility to use fatty acid metabolism to protect the heart under ischemic events represents an opportunity [43,288,289,290], but the protective effects may strictly depend on the fatty acid plasma levels of the patient [246]. Moreover, this kind of cardioprotective approach is controversial since cardiotoxicity, due to dangerous fatty acid intermediates, may occur [290].

## 6. Conclusions

The current knowledge regarding the strict relationship existing between cardiometabolism and human pathological conditions demonstrates that the ability of the metabolism to adapt, being flexible, is crucial for the physiological structure and function of the heart. Even if further and more specific studies are needed, it is evident that cardiac metabolic processes, and their changes under diseased conditions, may represent a promising therapeutic intervention in both acute and chronic heart pathologies. Together with the development of new pharmacological agents, a key issue is represented by treatment time-points and by the possibility to translate the therapy to pathophysiological conditions involving cardiometabolic changes. Overall, these data support the idea of metabolic therapy as an up-and-coming strategy in cardiac diseases.

## Figures and Tables

**Table 1:** Cardiometabolic changes in inherited cardiomyopathies.

Genetic Metabolic Cardiomyopathies	Cardiac Manifestations	Cardiometabolic Changes	References
Fatty acid oxidation disorders			
Carnitine deficiency	Dilated cardiomyopathy, cardiac arrest	Defective carnitine biosynthesis ↓ Fatty acid oxidation Lipid accumulation	[86,87,88,89,90,91,92,93,94,95]
Carnitine palmitoyltransferase II deficiency	Pansystolic murmur, septal hypertrophy, cardiac arrhythmias	Impaired mitochondrial acyl–CoA transport and fatty acid oxidation Cardiac lipidosis	[86,87,88,96,97,98]
Very-long-chain acyl-CoA dehydrogenase deficiency	Cardiac hypertrophy	↓ Fatty acid oxidation Cardiac lipidosis	[86,87,99,100,101]
Long-chain 3-hydroxyacyl-CoA dehydrogenase deficiency	Cardiac hypertrophy, cardiac arrhythmias	↓ Fatty acid oxidation Cardiac lipidosis	[86,87,102,103,104,105,106]
Mitochondrial trifunctional protein deficiency	Cardiac arrhythmias, conduction disorder, cardiorespiratory arrest	↓ Fatty acid oxidation Cardiac lipidosis	[86,87,107]
**Glycogen storage** **diseases**			
Glycogen storage diseases types II, III, IV and VI	Restrictive or dilated cardiomyopathies, conduction disorder	Glycogen accumulation Impaired autophagy and expression of mitochondrial genes	[86,107,108]
**Lysosomal storage** **disorders**			
Anderson–Fabry disease	Ventricular hypertrophy, valvular abnormalities, conduction disorder, cardiac arrhythmias	Globotriaosylceramide accumulation ↓ Activity of respiratory chain enzymes ↑ Oxidative stress ↑ Release of proinflammatory cytokines	[86,87,107,109,110,111]
Gaucher disease	Heart valve diseases	Glucosylceramide accumulation ↓ Activity of respiratory chain enzymes ↑ Oxidative stress ↑ Release of proinflammatory cytokines	[87,107,112,113]
Niemann–Pick disease	Endocardial fibroelastosis	Sphingomyelin accumulation ↓ Activity of respiratory chain enzymes ↑ Oxidative stress ↑ Release of proinflammatory cytokines	[87,107,114,115]
GM1 gangliosidosis	Heart failure	GM1-ganglioside accumulation ↓ Activity of respiratory chain enzymes ↑ Oxidative stress ↑ Release of proinflammatory cytokines	[87,107,109,110,112,114,116,117,118]
**Mitochondrial disorders**			
Friedreich ataxia	Cardiac hypertrophy, heart failure	↑ Oxidative stress Impaired mitochondrial respiratory function and iron metabolism	[86,107]
Barth syndrome	Dilated cardiomyopathy	↓ Electron transport chain activity ↓ Oxygen consumption ↑ Oxidative stress	[86,107]

**Table 2:** Cardiometabolic alterations in cardiac diseases.

Acute and Chronic Cardiac Diseases	Cardiometabolic Alterations	References
Ischemic heart disease	↓ Mitochondrial oxidative metabolism ↑ Utilization of glucose ↑ Rates of free fatty acid oxidation Accumulation of lactate and protons Reduction in intracellular pH	[138,139]
Heart failure	↑ Uptake of glucose and free fatty acid ↓ Uptake and oxidation of glucose and free fatty acid in mitochondria Cytosolic accumulation of metabolic intermediates Lipotoxicity and glucotoxicity ↑ Reliance on ketone bodies	[140]
Arrhythmias	Abnormalities in Ca^2+,^ K^+^, Na^+^ homeostasis Oxidative stress	[141]
Atrial fibrillation	↑ β-hydroxybutyrate generation, ketogenic amino acids (tyrosine and glycine) and 3-oxoacid-CoA transferase Mitochondrial dysfunction Oxidative stress	[142]
Hypertrophic cardiomyopathy	↓ Free fatty acid oxidation ↑ Ketone bodies and glucose oxidation	[143,144]
Dilated cardiomyopathy	↓ Oxidative metabolism in cardiomyocytes ↑ Anaerobic glycolysis ↑ Acylcarnitine and ketone bodies	[145]
Restrictive cardiomyopathy	Glycogen accumulation Amyloid deposits Iron overload Glycosphingolipids accumulation	[146]
Diabetic cardiomyopathy	↑ Free fatty acid release and myocyte sarcolemmal free fatty acid transporters. Lipotoxicity ↑ Triacylglycerols Impaired mitochondrial Ca^2+^ handling ↑ Oxidative stress	[147]
Valvular heart disease	↑ Lipid deposition and oxidized low-density lipoprotein formation ↑ Inflammation-associated factors ↑ Superoxide and hydrogen peroxide levels	[148]

**Table 3:** Metabolic pharmacological intervention in heart failure and myocardial infarction.

Cardiac Diseases	Pharmacological Intervention	Mechanism of Action	References
Heart Failure	Acipimox	Lipolysis inhibitor ↑ Glucose utilization	[191,192]
	Etomoxir, Perhexiline, and Oxfenicine	CPT-I inhibitors ↓ Fatty acid oxidation ↑ Glucose utilization	[2,193,194,195,196,197,198]
	Dichloroacetate	PDK inhibitor ↑ Glucose oxidation	[199,200,201,202]
	Thiazolidinediones	PPARγ agonists ↑ Glucose utilization	[206,207]
	SGLT2 inhibitors	↓ Na^+^/H^+^ exchanger activity ↑ Antioxidant ability of mitochondria	[208,209,210,211,212]
	Metformin	AMPK activator ↓ Oxidative stress and inflammation Improved endothelial function ↑ Glucose uptake	[218,219,220,221,222,223,224,225,226,227]
	GLP-1RAs	GLP-1 Receptor Analogues ↑ Glucose utilization	[229,230,231,232]
Myocardial infarction	AMPK activators	↑ Glycolytic pathway	[237,238,239]
	Aminooxyacetate and similar	Malate-aspartate shuttle inhibitors ↓ ROS production and oxidative stress	[240,241,242,243]
	Trimetazidine	3-KAT inhibitor ↓ Fatty acid oxidation ↑ Glucose utilization	[244,245,246,247]
	Dichloroacetate	PDK inhibitor ↑ Glucose oxidation	[248,249,250,251,252,253,254]
	GLP-1RAs	GLP-1 Receptor Analogues ↑ Glucose utilization	[255,256]
	GIK infusion	↑ Glucose metabolism ↓ Fatty acid utilization	[257,258,259,260]

## Data Availability

Not applicable.

## Abbreviations

AAC	acetyl-CoA carboxylase
ADP	adenosine diphosphate
AMP	adenosine monophosphate
AMPK	AMP-activated protein kinase
ANF	natriuretic factor
ATP	adenosine triphosphate
Ca2+	calcium
CABG	Coronary artery bypass graft surgery
CACT	carnitine acylcarnitine translocate
CAD	coronary artery disease
CPD	carnitine-palmitoyl transferase
CPT-I	carnitine palmitoyl transferase I
CPT-II	carnitine acyl-CoA transferase II
CVDs	cardiovascular diseases
FAODs	fatty acid oxidation disorders
G6P	glucose 6-phosphate
G6PDH	glucose 6-phosphate dehydrogenase
GFAT	fructose 6-phosphate amidotransferase
GLP-1	glucagon-like peptide-1
GLUT1	insulin-independent glucose transporter
GLUT4	insulin-sensitive glucose transporter
GSDs	glycogen storage diseases
GSK	glycogen synthase kinase
H+	protons
HBP	hexosamine biosynthetic pathway
HDL	high-density lipoproteins
HF	heart failure
h-FABP	heart-specific fatty acid-binding protein
I/R	ischemia/reperfusion
IEM	inborn errors of metabolism
IPC	ischemic preconditioning
K+	potassium
LCHAD	long chain 3-hydroxy-acyl-CoA dehydrogenase
LSDs	lysosomal storage disorders
MCD	malonyl-CoA decarboxylase
MI	myocardial infarction
mTOR	mechanistic Target of Rapamycin
ONOO–	peroxynitrite
PCD	primary carnitine deficiencies
PCI	primary percutaneous coronary intervention
PFK-1	phospho-fructokinase-1
PGC-1α	peroxisome proliferator-activated receptor-γ coactivator 1α
PH	primary hyperoxaluria
PPARα	peroxisome proliferator-activated receptor α
PPP	pentose phosphate pathway
PTP	permeability transition pore
ROS	reactive oxygen species
T2DM	type 2 diabetes mellitus
TGF-β	transforming growth factor β
UCP3	uncoupling protein 3
UDP-GlcNAc	uridine diphosphate-N-acetylglucosamine
VLCAD	very long-chain acyl-CoA dehydrogenase
WHO	World Health Organization
